# Lenacapavir with Fostemsavir in a Multidrug-Resistant HIV-Infected Hemodialysis Patient

**DOI:** 10.1155/2023/8865265

**Published:** 2023-10-18

**Authors:** Ferdinand Bigirimana, Sigi Van den Wijngaert, Christelle Fosso, Karolien Stoffels, Charlotte Martin, Evelyne Maillart, Philippe Clevenbergh

**Affiliations:** ^1^Infectious Diseases Department, University Hospital Brugmann, Université Libre de Bruxelles, Brussels, Belgium; ^2^Aids Reference Laboratory, Centre Hospitalier Universitaire St. Pierre, Brussels, Belgium; ^3^Nephrology and Dialysis Department, University Hospital Brugmann, Université Libre de Bruxelles, Brussels, Belgium; ^4^Infectious Diseases Department, University Hospital St Pierre, Université Libre de Bruxelles, Brussels, Belgium

## Abstract

We report a hemodialysis MDR HIV-infected patient switched to fostemsavir with lenacapavir plus lamivudine for more than a year. She maintained a suppressed viral replication and did not present any clinical or biological drug-related side effects. The combination of lenacapavir plus fostemsavir looks promising in terms of safety and efficacy even in patients with end-stage renal disease awaiting renal transplant. Both drugs are first in class ARVs so that there is no cross resistance with previous drugs, maintaining their efficacy against MDR HIV.

## 1. Introduction

HIV-infected patients with end-stage renal disease (ESRD) have limited therapeutic options, even more if they harbor antiretroviral (ARV)-resistant HIV strains. Lenacapavir (LEN) [1] and fostemsavir (FTR) [2] are two recently approved ARVs combining activity against multidrug-resistant HIV, a possible administration in patients receiving hemodialysis or peritoneal dialysis, and a manageable drug-drug interactions (DDIs), for example, with antirejection medication in the case of renal transplantation. They have no cross-resistance with previous ARV classes and could be administered together, without anticipated mutual DDI.

Fostemsavir (FTR) is a first-in-class prodrug metabolized to temsavir (TMR) by alkaline phosphatase on the luminal surface of the small intestine. It binds to the gp120 subunit of HIV-1, thus preventing viral attachment and cellular entry [3]. Temsavir pharmacokinetics may be affected by drugs inducing or inhibiting P-glycoprotein, CYP3A enzymes, esterases, and/or breast cancer-resistant protein (BCRP) [4]. However, pharmacokinetics (PKs) interaction studies with other ARVs (ritonavir, cobicistat, and NNRTI) showed no significant changes in TMR concentrations when coadministered with strong inhibitors or moderate inducers of CYP3A4, P-gp, or BCRP [4]. On the contrary, TMR has no effect on CYP3A4 and anticipated weak DDI with other drugs including tacrolimus [5]. TMR may increase the level of certain direct anti HCV drugs by organic anion transporting polypeptide (OATP)1B1/3 inhibition. TMR PK is not altered in ESDR patients including those on hemodialysis [6].

Lenacapavir (LEN), a first-in-class drug, is an HIV capsid inhibitor. It interferes at multiple stages, thus disrupting the viral multiplication cycle with a prolonged duration of action [1]. Lenacapavir is administered with an oral induction phase followed by a maintenance subcutaneous dose every 6 months. No dosage adjustment of lenacapavir is recommended in patients with mild, moderate, or severe renal impairment. There are no data in patients with ESRD. As LEN is highly protein bound, no effect of hemodialysis (HD) is expected. It is mainly cleared unchanged in the stools, with less than 1% excreted by the urine. It is a substrate of P-gp, UGT1A1, and CYP3A; hence, inducers or inhibitors of those enzymes seriously affect the PK of LEN. LEN itself is a moderate inhibitor of CYP3A affecting coadministered medication [7]. There is a potential increase in several drugs' levels, including tacrolimus.

There is no report of the use of LEN in association with FTR in ESRD HIV-infected patients in terms of efficacy and safety. We report here the first HIV-infected hemodialysis patient treated with LEN/FTR/lamivudine.

### 1.1. Ethical Aspects

The patient has given her written informed consent for publication. Lenacapavir is available through a compassionate access program by Gilead.

## 2. Case Report

This is a 35-year-old woman, vertically infected by HIV-1, in CDC stage C3 (esophageal candidiasis and disseminated TB). There is no HBV or HCV coinfection. She received sequentially all available ARVs since her early childhood and developed treatment failure due to multiresistance associated with bursts of nonadherence. Furthermore, the patient developed HIV-associated nephropathy with ESDR requiring peritoneal dialysis (09/2016) and hemodialysis (09/2020), awaiting renal transplantation. The patient was transplanted in August 2023. Other comorbidities are secondary hyperparathyroidism, pathological hip fracture, and abacavir allergy. Her cumulative genotypic resistance profile (dated 03.2021) combines all the resistance-associated mutations observed over time (Table 1). They were for NRTI 41L, 67N, 69D, 70R, 184V, 215F/V/L, 219Q; for NNRTI, 103N, 138A, 179I, 181C, 225H; for PI 10I/F, 11I, 20R, 32I, 33F, 43T, 54V/L, 74P, 84V, 90M; and for INSTI 97A, 143R [8] (Table 1).

## 3. Discussion

We report an ESRD MDR HIV-infected patient switched to FTR with LEN plus lamivudine for more than a year. She maintained a suppressed viral replication and did not present any clinical or biological drug-related side effects (Table 2).

There is no switch study using FTR or LEN in virally suppressed HIV-infected patients or a study combining these two drugs yet. However, in our patient, the number of daily tablets dramatically dropped from 7 to 3 combined with biannual subcutaneous injections. Two drugs regimens are now common in order to avoid NRTIs or boosted-PI [10]. LEN plus optimal backbone ARVs (OBR) has shown superior virological efficacy compared to other regimens, in particular FTR + OBR, in heavily treatment-experienced (HTE) patients failing their regimen [11]. As experienced by our patient, the most prevalent side effect of LEN is injection site pain and induration. TMR is associated with metabolic abnormalities (glucose and lipid levels) in 4-5% of the patients. Decrease in GFR in some patients did not appear to be FTR related. Our patient did not experience significant changes in liver enzymes, glucose, or lipid levels. After subcutaneous administration, LEN has a half-life of 8–12 weeks, the first real long-acting ARV. Search for companion drugs to coadminister with LEN is in progress, like the ultra-long-acting parenteral prodrug formulation of dolutegravir [12]. A biannual subcutaneous two drugs' regimen could be a true game changer for adherence [13]. Costs and drug's availability remain an issue. A preprint has previously been published [14].

## 4. Conclusion

The combination of lenacapavir and fostemsavir is a viable treatment option for highly treatment-experienced patients either as a rescue regimen or as a switch regimen. It decreases the pill burden and the potential for drug-drug interactions. Lenacapavir and/or fostemsavir appear to be safe in end-stage renal disease patients including those on hemodialysis. Metabolic side effects and effect on weight have still to be evaluated. Prospective studies using this combination are required as rescue, switch, or even first-line therapy.

## Figures and Tables

**Table 1:** Evolution of the viral resistances according to time.

Sample dates	28 December 2005	31 January 2007	11 January 2008	13 September 2008	14 January 2009	26 July 2010	20 October 2011	09 September 2016	19 May 2017	05 March 2021	Cumulative
Viral load (copies/mL)	67000	>100000	>100000	31000	63300	34300	563	572000	398000	20200	
CD4 count (/*μ*L)	488	109	141	203	173	383	336	11	13	591	

*NRTI*											
Lamivudine (3TC)	ILL	S	ILL	R	—	—	ILL	S	R	R	R
Abacavir (ABC)	R	IR	R	R	—	—	R	ILL	IR	IR	R
Zidovudine (AZT)	R	R	R	R	—	—	R	IR	IR	R	R
Stavudine (D4T)	R	R	R	R	—	—	R	IR	IR	R	R
Didanosine (DDI)	R	R	R	R	—	—	R	IR	R	R	R
Emtricitabine (FTC)	ILL	S	ILL	R	—	—	ILL	S	R	R	R
Tenofovir (TDF)	R	IR	R	IR	—	—	R	ILL	S	SP	R

*NNRTI*											
Doravirine (DOR)	S	S	S	S	—	—	SP	IR	IR	ILL	IR
Efavirenz (EFV)	S	S	S	S	—	—	IR	R	R	R	R
Etravirine (ETR)	S	S	S	S	—	—	IR	IR	IR	IR	IR
Nevirapine (NVP)	S	S	S	S	—	—	R	R	R	R	R
Rilpivirine (RPV)	S	S	S	S	—	—	IR	R	R	IR	R

*PI*											
Atazanavir (ATV/r)	R	S	R	R	—	—	R	R	R	R	R
Darunavir (DRV/r)	ILL	S	ILL	ILL	—	—	ILL	R	R	R	R
Fosamprenavir (FPV/r)	R	S	R	R	—	—	R	R	R	R	R
Indinavir (IDV/r)	R	S	R	R	—	—	R	R	R	R	R
Lopinavir (LPV/r)	R	S	R	R	—	—	R	R	R	R	R
Nelfinavir (NFV)	R	S	R	R	—	—	R	R	R	R	R
Ritonavir (/r)	R	S	R	R	—	—	R	R	R	R	R
Saquinavir (SQV/r)	R	S	R	R	—	—	R	R	R	R	R
Tipranavir (TPV/r)	R	S	R	R	—	—	R	R	R	R	R

*INSTI*											
Bictegravir (BIC)	—	—	—	—	—	S	S	SP	SP	SP	SP
Cabotegravir (CAB)	—	—	—	—	—	S	S	ILL	ILL	ILL	ILL
Dolutegravir (DTG)	—	—	—	—	—	S	S	SP	SP	SP	SP
Elvitegravir (EVG)	—	—	—	—	—	S	S	IR	IR	IR	IR
Raltegravir (RAL)	—	—	—	—	—	S	S	R	R	R	R

*Attachment inhibitors*											
Maraviroc (MVC)	—	—	—	—	CXCR4 use (phenotypic)	—	—	—	CCR5 use (FPR 17%)	CXCR4 use (FPR 0.1%)	—

INSTI: integrase strand transfer inhibitor, NNRTI: non-nucleoside reverse transcriptase inhibitors, NRTI: nucleoside reverse transcriptase inhibitors, PI: protease inhibitor; ILL: low-level resistance, IR: intermediate resistance, R: high-level resistance, S: susceptible, SP: potential low-level resistance, —: result not available. HD: hemodialysis, PD: peritoneal dialysis, eGFR: estimated glomerular filtration rate, IU: international unit, PA: alkaline phosphatase, Hb: hemoglobin, cp/ml: copies/milliliter, SC: subcutaneous.

**Table 2:** Antiretroviral treatment and biological data history.

Date	CD4 (cells/*μ*l)	Viral load (cp/ml)	Regimen	Biochemistry
05/2017	13	398.000	Darunavir/ritonavir, lamivudine	Glucose: 93 mg/dL, total cholesterol: 184 mg/dL, eGFR: 7 ml/min, GOT: 26 IU/ml, GPT: 9 IU/ml, PA: 102 IU/ml, GGT: 41 IU/ml, Hb: 6.6 g/dl, platelets: 82 × 10^3^/*μ*L, weight: 45 kg, PD
06/2017	47	1430	Induction with foscarnet [9] followed by SC enfuvirtide, maraviroc, tenofovir, lamivudine, high dose darunavir/r, high dose dolutegravir	Glucose: NA, total cholesterol: NA, eGFR: 7 ml/min, GOT: 16 IU/ml, GPT: 18 IU/ml, PA: 289 IU/ml, GGT: 112 IU/ml, Hb: 9.5 g/dl, platelets: 283 × 10^3^/*μ*L, weight: 45.4 kg, PD
12/2017	379	<20	SC enfuvirtide, maraviroc, tenofovir, lamivudine, high dose darunavir/r, high dose dolutegravir	Glucose: 224 mg/dL total cholesterol: 139 mg/dL, eGFR: 6 ml/min, GOT: 12 IU/ml, GPT: 160 IU/ml, PA: 235 IU/ml, GGT: 55 IU/ml, Hb: 9.20 g/dl, platelets: 252 × 10^3^/*μ*L, weight: NA, PD
04/2018	348	<20	Idem stop tenofovir 12/2018	Glucose: 130 mg/dL, total cholesterol: 134 mg/dL, eGFR: 5 ml/min, GOT: 10 IU/ml, GPT: 9 IU/ml, PA: 209 IU/ml, GGT: 35 IU/ml, Hb: 10.2 g/dl, platelets: 2 64 × 10^3^/*μ*L, weight: 52 kg, PD
09/2019	582	<20	SC enfuvirtide, maraviroc, lamivudine, high dose darunavir/r, high dose dolutegravir	Glucose: 111 mg/dL, total cholesterol: NA, eGFR: 5 ml/min, GOT: 17 IU/ml, GPT: 17 IU/ml, PA: 689 IU/ml, GGT: 41 IU/ml, Hb: 9.3 g/dl, platelets: 287 × 10^3^/*μ*L, weight: 51.9 kg, PD
04/2020	570	<20	SC enfuvirtide, maraviroc, lamivudine, high dose darunavir/r, high dose dolutegravir	Glucose: 98 mg/dL, total cholesterol: 176 mg/dL, eGFR: 6 ml/min, GOT: 10 IU/ml, GPT: 6 IU/ml, PA: 726 IU/ml, GGT: 19 IU/ml, Hb:10.3 g/dl, platelets: 270 × 10^3^/*μ*L, weight: 39.5 kg, PD
09/2020	456	37	Stop enfuvirtide 02/2020	HD
03/2021	591	20200	Nonadherence, resumption of same treatment + counselling	03/2021 genotypic resistance testing: results shown above
10/2021	429	338	Maraviroc, lamivudine, high dose darunavir/r, high dose dolutegravir	Glucose: 130 mg/dL, Hb A1c: 6.1%, total cholesterol: 98 mg/dL, triglyceride:84 mg/dL, eGFR: 4 ml/min, GOT: 28 IU/ml, GPT: 17 IU/ml, PA: 105 IU/ml, GGT: 17 IU/ml, Hb: 10.6 g/dl, platelets: 178 × 10^3^/*μ*L,weight: 48.9 kg, HD
06/2022	486	<20	LEN: oral loading dose over 8 days then 2 subcutaneous injections of 463.5 mg/1.5 mL biannually + FTR: 600 mg BID + 3TC: 75 mg QD	Glucose: 82 mg/dL, total cholesterol: 117 mg/dL, triglyceride: 74 mg/dL, eGFR:4 ml/min, GOT: 15 IU/ml, GPT: 11 IU/ml, PA: 112 IU/ml, GGT: 14 IU/ml, Hb: 11.8 g/dl, platelets: 244 × 10^3^/*μ*L,weight: 53.5 kg, HD
12/2022	388	<20	LEN + FTR + 3TC	Glucose: 196 mg/dL, Hb A1c: 5.4%, total cholesterol: 115 mg/dL, Triglyceride: 58 mg/dL, eGFR: 4 ml/min, GOT: 18 IU/ml, GPT: 14 IU/ml, PA: 126 IU/ml, GGT: 15 IU/ml, Hb: 12 g/dl, platelets: 149 × 10^3^/*μ*L, weight: 53.5 kg, HD
07/2023	392	<20	LEN + FTR + 3TC	Glucose: 190 mg/dL, HbA1c: 5.3%, total cholesterol: 105 mg/dL, triglyceride: 51 mg/dL, eGFR: 3 ml/min, GOT: 17 IU/ml, GPT: 13 IU/ml, PA: 152 IU/ml, GGT: 21 IU/ml, Hb: 12.5 g/dl, platelets: 195 × 10^3^/*μ*L, weight: 52.5 kg, HD
08/2023			LEN + FTR + 3TC	Renal transplantation

## Data Availability

The data used to support the findings of this study are available on request from the corresponding author.

## References

[1] Tailor M. W., Chahine E. B., Koren D., Sherman E. M. (2023). Lenacapavir: a novel long-acting capsid inhibitor for HIV. *The Annals of Pharmacotherapy*.

[2] Kozal M., Aberg J., Pialoux G. (2020). Fostemsavir in adults with multidrug-resistant HIV-1 infection. *New England Journal of Medicine*.

[3] Seval N., Frank C., Kozal M. (2021). Fostemsavir for the treatment of HIV. *Expert Review of Anti-infective Therapy*.

[4] Moore K., Thakkar N., Magee M. (2022). Pharmacokinetics of temsavir, the active moiety of the HIV-1 attachment inhibitor prodrug, fostemsavir, coadministered with cobicistat, etravirine, darunavir/cobicistat, or darunavir/ritonavir with or without etravirine in healthy participants. *Antimicrobial Agents and Chemotherapy*.

[5] Hiv drug interactions (2023). University of liverpool. https://www.hiv-druginteractions.org/drug_queries/785171/drug_query_interactions.

[6] Highlights of prescribing information (2023). Rukobia (fostemsavir) extended-release tablets, for oral use. https://gskpro.com/content/dam/global/hcpportal/en_US/Prescribing_Information/Rukobia/pdf/RUKOBIA-PI-PIL.PDF.

[7] Highlights of prescribing information (2023). SUNLENCA® (lenacapavir) tablets, for oral use and SUNLENCA® (lenacapavir) injection, for subcutaneous use. https://www.gilead.com/-/media/files/pdfs/medicines/hiv/sunlenca/sunlenca_pi.pdf.

[8] SmartGene (2022). Multi-drug resistant panels. https://hivdb.stanford.edu/IDNS®version.v3_12_0r1(r32526)/Stanford.HIVDB.

[9] Delory T., Papot E., Rioux C. (2016). Foscarnet, zidovudine and dolutegravir combination efficacy and tolerability for late stage HIV salvage therapy: a case‐series experience. *Journal of Medical Virology*.

[10] Sivanandy P., Ng Yujie J., Chandirasekaran K., Hong Seng O., Azhari Wasi N. A. (2023). Efficacy and safety of two-drug regimens that are approved from 2018 to 2022 for the treatment of human immunodeficiency virus (HIV) disease and its opportunistic infections. nur azrida azhari wasi. *Microorganisms*.

[11] Chatzidaki I., Curteis T., Luedke H. (2023). Indirect treatment comparisons of lenacapavir plus optimized background regimen versus other treatments for multidrug-resistant human immunodeficiency virus. *Value in Health*.

[12] Deodhar S., Sillman B., Bade A. N. (2022). Transformation of dolutegravir into an ultra-long-acting parenteral prodrug formulation. *Nature Communications*.

[13] Nachega J. B., Scarsi K. K., Gandhi M. (2023). Long-acting antiretrovirals and HIV treatment adherence. *The Lancet HIV*.

[14] Clevenbergh P., Bigirimana F., Wijngaert S. V. D. (2023). Lenacapavir with Fostemsavir in a multi-drug resistant HIV-infected hemodialysis patient. https://europepmc.org/article/ppr/ppr699506.

